# Sex Differences in Reported Adverse Drug Reactions to Angiotensin-Converting Enzyme Inhibitors

**DOI:** 10.1001/jamanetworkopen.2022.8224

**Published:** 2022-04-20

**Authors:** Sophie H. Bots, Michelle M. Schreuder, Jeanine E. Roeters van Lennep, Sarah Watson, Eugène van Puijenbroek, N. Charlotte Onland-Moret, Hester M. den Ruijter

**Affiliations:** 1 Laboratory for Experimental Cardiology, University Medical Center Utrecht, Utrecht University, Utrecht, the Netherlands; 2 Department of Internal Medicine, Vascular Medicine, Erasmus Medical Center, Rotterdam, the Netherlands; 3 Uppsala Monitoring Centre, Uppsala, Sweden; 4 Pharmacovigilance Centre Lareb, ‘s-Hertogenbosch, the Netherlands; 5 Groningen Research Institute of Pharmacy, PharmacoTherapy, Epidemiology and Economics, University of Groningen, Groningen, the Netherlands; 6 Julius Center for Health Sciences and Primary Care, University Medical Center Utrecht, Utrecht, the Netherlands

## Abstract

This cross-sectional study investigates differences by sex in reporting of adverse drug reactions associated with angiotensin-converting enzyme inhibitors combining global and prescription-corrected databases.

## Introduction

Sex differences in adverse drug reactions (ADRs) associated with angiotensin-converting enzyme inhibitors (ACEIs) remain poorly understood owing to a lack of sex-specific ADR data from clinical trials.^1^ Postmarketing pharmacovigilance data, containing structured and detailed ADR information, may play an important role in such analyses. However, these data are often not corrected for prescription numbers and therefore cannot separate sex differences in ADR risk from sex differences in prescription rates. To investigate whether women report more ACEI-related ADRs than men after correction for sex-specific prescription and describe sex differences in reported ADR types, we combined data from the global pharmacovigilance database VigiBase and the prescription-corrected Dutch pharmacovigilance database Lareb.

## Methods

We studied all ADR reports submitted by patients and health care professionals between 1980 and January 2020 for VigiBase and 2003 and January 2021 for Lareb that included information on sex. Drug name, patient sex and age, and detailed ADR classification were extracted. Outcomes were number of reports by sex and type of ADR classified according to MedDRA hierarchy. Dutch prescription data were obtained from the Medical Product Information Project database. Sex-specific reporting rates of ADRs per 100 000 individuals were calculated by dividing the total number of reports by the total number of individuals. We used rate differences and incidence rate ratios to investigate whether sex differences in ADR incidence were statistically significant. We calculated and compared the ADR type-specific number of and absolute difference in reports (eMethods in the Supplement).

## Results

VigiBase included 227 482 ACEI-related ADR reports (53% women), and Lareb included 3903 reports (52% women). Most reports came from individuals aged 45 to 64 years (98 339 individuals [42.5%]). After Lareb data were corrected for sex-specific prescription rates, the ADR reporting rate per 100 000 individuals was 25 reports in women and 18 reports in men, for an absolute rate difference of 6 reports (95% CI, 4 to 7 reports) per 100 000 individuals. Women had a 1.31-fold higher reporting rate of ADRs (95% CI, 1.27-1.35) compared with men. Cough and angioedema were the most frequently reported ADRs among women and men in VigiBase and Lareb (Table). Women outnumbered men in 19 of 27 ADR categories, with most reports with more women in respiratory, gastrointestinal, and general disorders categories and reports with more men in skin and subcutaneous tissue, kidney and urinary, and reproductive system and breast tissue disorder categories (Figure, A). Figure, B-G, shows a more detailed breakdown across ADR types within 3 categories with the largest excess of female reports (B-D) and male reports (E-G).

**Table.** Most Commonly Reported ADRs by Sex^a^

ADR rank by frequency of reporting	ADR type (No. of reports)			
	VigiBase		Lareb	
	Women	Men	Women	Men
1	Cough (10 909)	Cough (7701)	Angioedema (199)	Angioedema (153)
2	Angioedema (3441)	Angioedema (6634)	Cough (163)	Cough (124)
3	Dizziness (2509)	Acute kidney injury (2830)	Therapeutic response unexpected (70)	Therapeutic response unexpected (91)
4	Drug hypersensitivity (2323)	Hyperkalemia (2159)	Dizziness (51)	Dizziness (49)
5	Headache (1965)	Dizziness (2056)	Headache (51)	Pruritus (45)
6	Nausea (1810)	Hypotension (1949)	Alopecia (44)	Erectile dysfunction (37)
7	Acute kidney injury (1793)	Dyspnea (1540)	Dyspnea (42)	Fatigue (36)
8	Dyspnea (1723)	Pruritus (1309	Nausea (39)	Myalgia (29)
9	Drug ineffective (1605)	Drug ineffective (1272)	Paresthesia (37)	Headache (29)
10	Pruritus (1456)	Headache (1213)	Rash (35)	Muscle spasms (27)

Abbreviation: ADR, adverse drug reaction.

^a^ ADRs are collected at the MedDRA preferred term level in the global database VigiBase and the Dutch database Lareb.

**Figure.**
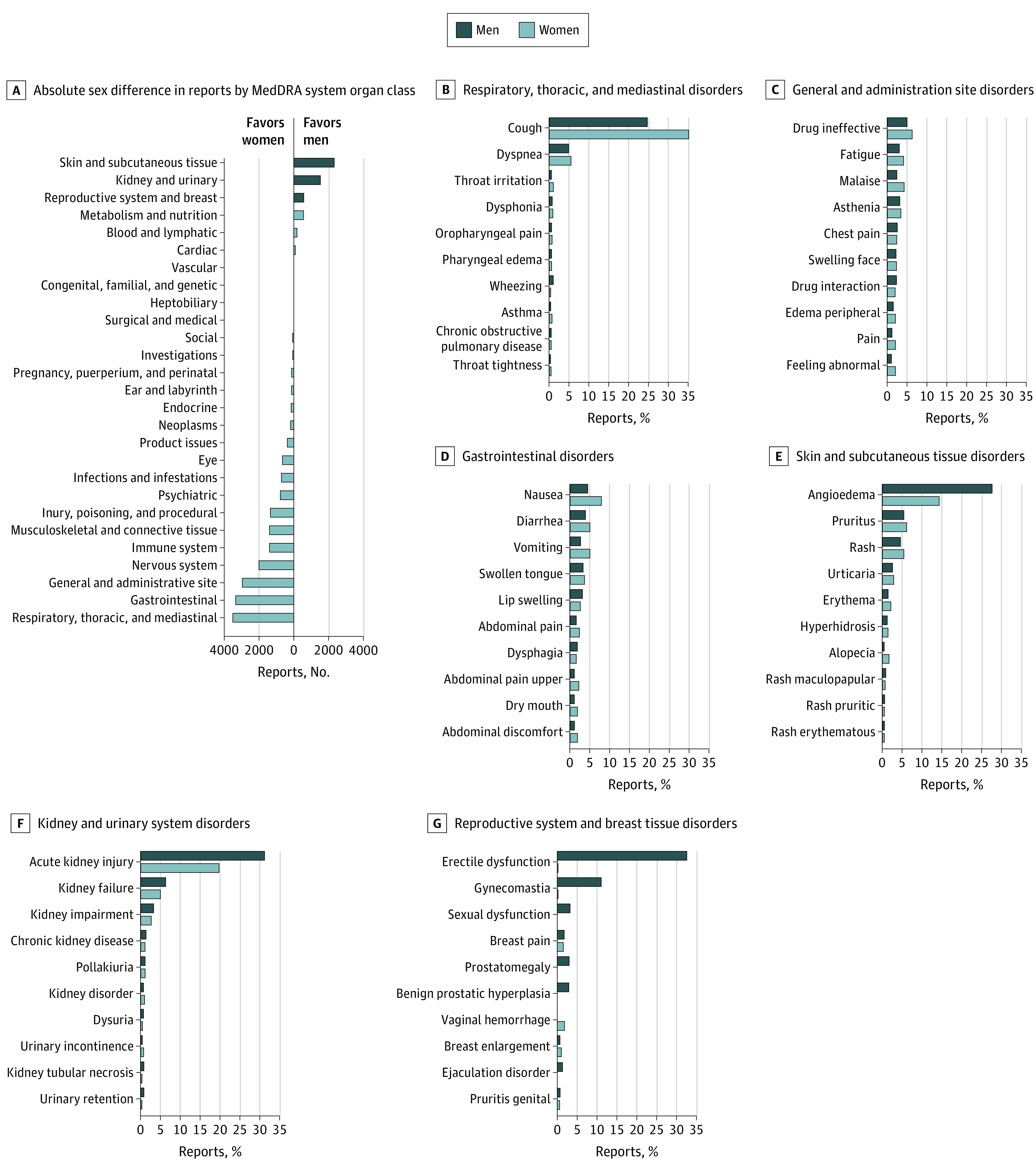
Adverse Drug Reaction Reports From Women and Men

## Discussion

These findings are in line with a previous study^2^ suggesting that women report more ADRs than men. The 1.31-fold higher ADR reporting in women compared with men is large considering that ACEIs comprise one of the first-line treatments of choice for cardiovascular conditions common in women and men, such as hypertension.^3^ Given that ADRs play an important role in adherence^4^ and failure to reach guideline-recommended target doses, sex-stratified comparison trials equally powered for women and men are needed to explore whether different dosages or ACEI alternatives are associated with decreased ADR risk. These studies should give priority to ADRs associated with the greatest differences in adherence, which our study and previous literature^5^ suggest may differ by sex. Importantly, we may have underestimated ADR incidence owing to underreporting.^6^ Our 95% CIs may be artificially narrow because we could not account for in-person clustering of reports. In addition, our findings need validation in specific settings given that country-specific prescription practices or comorbidities may be associated with ADR risk and reporting differences.

Our study provides evidence for sex differences in ACEI-related ADRs, with women reporting more ADRs and different types of ADRs compared with men. These findings suggest the need for further studies to elucidate mechanisms underlying women’s higher reporting rates and optimal treatment strategies for women and men.

## Notes

Supplement.
eMethods.

eReferences.
