# Supplementary material for: Sex Differences in Reported Adverse Drug Reactions to Angiotensin-Converting Enzyme Inhibitors
Source: JAMA Netw Open. 2022 Apr 20;5(4):e228224. doi: 10.1001/jamanetworkopen.2022.8224 (PMC9021909; doi:10.1001/jamanetworkopen.2022.8224)
Supplement: Supplement. — eMethods. eReferences. [file jamanetwopen-e228224-s001.pdf]

Supplemental Online Content

Bots SH, Schreuder MM, Roeters van Lennep JE, et al. Sex differences in reported adverse drug reactions to angiotensin-converting enzyme inhibitors. *JAMA Netw Open*. 2022;5(4):e228224. doi:10.1001/jamanetworkopen.2022.8224

eMethods.

eReferences.

This supplemental material has been provided by the authors to give readers additional information about their work.

© 2022 Sophie H. Bots et al. *JAMA Network Open.*

eMethods.

Study databases

We used pharmacovigilance data from the global database VigiBase[1] and the Dutch database Lareb[2]. We obtained Dutch prescription data from the Genees- en hulpmiddelen Informatie Project (GIP) database[3]. As these data were anonymised, ethical approval for the study was waived and informed consent was not required. Importantly, these organisations collect information at the prescription level with the aim of post-marketing safety monitoring of medications. Therefore, information on dosage, indication, and patient-level characteristics such as concomitant medication use is often not registered or registered incompletely.

Vigibase

VigiBase collects ADR reports shared between member countries of the WHO Programme for International Drug Monitoring, submitted by both healthcare professionals and, in some countries, directly from patients. Reports can also be submitted by manufacturing pharmaceutical companies. For the current study, we used all reports on ACEIs collected between their introduction to the market in the 1980s and January 2020. Reports without information on sex were excluded and suspected duplicate reports were removed using VigiMatch[4], the duplicate detection method developed at the Uppsala Monitoring Centre.

We extracted detailed ADR data for the five individual ACEI medications with the largest number of serious ADR reports and five with the largest number of non-serious ADR reports. We extracted the name of the drug, sex of the person who experienced the ADR, seriousness of the ADR, and the complete classification of the ADR type according to MedDRA. The ADR reports were classified as serious if they resulted in death, were life-threatening, caused or prolonged hospitalisation, resulted in permanent disability, caused congenital anomalies or birth defects, or resulted in other medically important conditions. We also extracted the number of ADR reports per age group.

Lareb and GIP database

The Lareb collects ADR reports from patients, healthcare professionals and marketing Authorisation Holders in the Netherlands. The GIP database tracks the number of people that use certain medications in the Netherlands based on claims from 24 health insurance companies. Together, these companies cover almost 17 million people, which is close to the entire population of the Netherlands. It covers all medications insured by the standard medical care insurance, which each person living and working in the Netherlands must have by law[3].

For the current study, we extracted all ACEI-reports collected by the Lareb between 1 January 2003 and 1 January 2021 because GIP data was not available before 2003. Reports without

© 2022 Sophie H. Bots et al. *JAMA Network Open.*

information on sex or from patients younger than 25 years were excluded because these were unlikely to suffer from cardiovascular disease. Reports submitted by pharmaceutical industry were also excluded as it could not be certified if these derived from patients treated in the Netherlands. We extracted detailed ADR information for all ACEI-related reports that fell within the selected time period. We extracted the name of the drug, sex and age of the person who experienced the ADR, and the classification of the ADR type according to MedDRA. We extracted the number of individual users that were prescribe ACEIs stratified by year, sex, and 10-year age categories.

Statistical analysis

We calculated the sex-specific total number of reports stratified by ADR preferred term code according to the MedDRA system. This is a hierarchical system that groups similar ADR types together at several levels of detail, with the Preferred Term (PT) level being most detailed level available and the System Organ Classes (SOC) level being least detailed. To compare ADR types between the sexes, the absolute sex difference in number of reports for each primary SOC was calculated. Results are given as numbers with percentages or bar graphs.

The sex-specific number of ADRs per 100,000 users was calculated by dividing the total number of ADR reports by the total number of prescriptions for each sex separately. We considered this to be an incidence rate, as the number of cases (ADRs) could be larger than the number of people at risk (prescriptions). To test whether the difference in ADR incidence was statistically significant, we calculated the rate difference and incidence rate ratio with 95% confidence interval. A p-value < 0.05 was considered statistically significant.

All analyses were performed in R (R Core Team, Vienna, Austria). We follow the STROBE reporting guidelines for cohort studies.

© 2022 Sophie H. Bots et al. *JAMA Network Open.*

eReferences.

1. Uppsala Monitoring Centre. VigiBase. 2021. https://www.who-umc.org/vigibase/vigibase/ (accessed 01 February 2021).
2. Bijwerkingencentrum Lareb. Wat is Bijwerkingencentrum Lareb. 2021. https://www.lareb.nl/pages/wat-is-bijwerkingencentrum-lareb.
3. Zorginstituut Nederland. Toelichting GIPdatabank. 2021. https://www.gipdatabank.nl/servicepagina/toelichting.
4. Norén GN, Orre R, Bate A, Edwards IR. Duplicate detection in adverse drug reaction surveillance. *Data Mining and Knowledge Discovery* 2007; **14**(3): 305-28.

© 2022 Sophie H. Bots et al. *JAMA Network Open.*
